# Effect of Diabetes Online Community Engagement on Health Indicators: Cross-Sectional Study

**DOI:** 10.2196/diabetes.8603

**Published:** 2018-04-24

**Authors:** Michelle L Litchman, Linda S Edelman, Gary W Donaldson

**Affiliations:** 1 College of Nursing University of Utah Salt Lake City, UT United States; 2 Utah Diabetes and Endocrinology Center Salt Lake City, UT United States; 3 Department of Anesthesiology University of Utah Salt Lake City, UT United States

**Keywords:** diabetes, online peer support, social media, eHealth, quality of life, self-care, A1c

## Abstract

**Background:**

Successful diabetes management requires ongoing lifelong self-care and can require that individuals with diabetes become experts in translating care recommendations into real-life day-to-day diabetes self-care strategies. The diabetes online community comprises multiple websites that include social media sites, blogs, and discussion groups for people with diabetes to chat and exchange information. Online communities can provide disease-specific practical advice and emotional support, allow users to share experiences, and encourage self-advocacy and patient empowerment. However, there has been little research about whether diabetes online community use is associated with better diabetes self-care or quality of life.

**Objective:**

The aim of this study was to survey adults with diabetes who participated in the diabetes online community to better understand and describe who is using the diabetes online community, how they are using it, and whether the use of the diabetes online community was associated with health indicators.

**Methods:**

We recruited adults diagnosed with diabetes who used at least one of 4 different diabetes-related online communities to complete an online survey. Participants’ demographics, reported glycated hemoglobin (HbA_1c_), health-related quality of life (SF-12v2), level of diabetes self-care (Self-Care Inventory-Revised), and diabetes online community use (level of intensity and engagement) were collected. We examined the relationships between demographics, diabetes online community use, and health indicators (health-related quality of life, self-care, and HbA_1c_ levels). We used binary logistic regression to determine the extent to which diabetes online community use predicted an HbA_1c_ <7% or ≥7% after controlling statistically for other variables in the model.

**Results:**

A total of 183 adults participated in this study. Participants were mostly female (71.6%, 131/183), white (95.1%, 174/183), US citizens (82.5%, 151/183), had type 1 diabetes (69.7%, 129/183), with a mean age of 44.7 years (SD 14) and diabetes duration of 18.2 years (SD 14.6). Participants had higher diabetes self-care (*P*<.001, mean 72.4, SD 12.1) and better health-related quality of life (physical component summary *P*<.001, mean 64.8, SD 19; mental component summary *P*<.001, mean 66.6, SD 21.6) when compared with norms for diabetes. Diabetes online community engagement was a strong predictor of A_1c_, reducing the odds of having an A_1c_ ≥7% by 33.8% for every point increase in diabetes online community engagement (0-5). Our data also indicated that study participants are oftentimes (67.2%, 123/183) not informing their healthcare providers about their diabetes online community use even though most (91.2%, 161/181) are seeing their healthcare provider on a regular basis.

**Conclusions:**

Our results suggest that individuals highly engaged with diabetes online community are more likely to have better glycemic levels compared with those with lower engagement. Furthermore, diabetes online community users have high health-related quality of life and diabetes self-care levels. Supplementing usual healthcare activities with diabetes online community use may encourage knowledge and support among a population that needs to optimize its diabetes self-care. Further studies are needed to determine how diabetes online community engagement may affect health outcomes.

## Introduction

### Background

The internet is increasingly used as a source of health information. In fact, 79% of adults in the United States use the internet and, of those, 59% are looking for health information [1]. It is observed that 23% of individuals with chronic conditions look online to find someone with similar health concerns [2]. Websites that allow interaction and crowdsourcing the collective wisdom of others [3] can help patients manage their own health by providing tools for health promotion and disease self-care, decision support, support for behavior change, and access to online communities [4]. Online communities can support health literacy by crowdsourcing information to support medical decision making [5,6]. Although many patients are using online information and communities to improve health [7] and engagement, how online activity affects health outcomes is poorly understood, and measuring meaningful eHealth engagement can be difficult [8].

As diabetes is a complex condition, some people with diabetes find patient peers helpful in providing support for managing their disease. Engagement in peer health is defined as the interaction, education, and support offered by peers with the same condition to promote self-care. Peers who receive special training can provide assistance in day-to-day chronic disease management, encourage appropriate clinical care, and offer ongoing social and emotional support [9,10]. Trained peers with diabetes have provided formal face-to-face support or discussion groups [10,11], phone calls [10,12,13], text support, and home visits [10]. Diabetes-related peer health has been associated with increased knowledge [14,15], self-efficacy [11,12,16], patient activation [11,16], communication with physicians, healthier eating habits [11,17], and improved hemoglobin A_1c_ [13,15-24]. Importantly, reciprocal peer support has been found to be better than nurse care management with regard to glycated hemoglobin (HbA_1c_) reduction [13]. The American Diabetes Association and American Association of Diabetes Educators recognize peers as an important factor in diabetes self-care [25,26]. However, the informal or unstructured peer support provided by the diabetes online community (DOC) has not been fully addressed.

### Diabetes Online Community

The DOC is a grassroots online community developed for the purpose of sharing knowledge and support based on the user’s experience of living with diabetes. Individuals involved in the DOC were initially those living with diabetes themselves, although the community has expanded and now includes family, friends, healthcare providers, and industry representatives [27]. DOC provides a vehicle for individuals to learn practical diabetes self-care techniques from experienced peers with shared experiences [27-29], and can be a source of confidence in diabetes self-care [30], inspiration, motivation, and encouragement [31], all of which support health literacy. The DOC includes blogs, video vlogs, discussion boards, and diabetes-specific (ie, Reality Check, TuDiabetes, Diabetic Connect, Beyond Type 1, Diabetes Daily) and general social media sites (ie, Facebook, Twitter, Instagram).

The DOC can be accessed through stand-alone interactive websites or social media sites. DOC users can actively contribute to discussions or passively view posts without contributing to the discussion [32]. DOC users engage in peer health [33,34] to gain practical advice [35-38], emotional support [35-41], shared experience [37-39], and improved coping [42] and empowerment [40]. There is limited data on negative patient outcomes related to DOC use [43]; however, misinformation on social media sites is infrequent [35,36,44,45] and quickly corrected by other members in the discussion group [35,39].

Currently, we are not aware of any research examining the relationship between DOC engagement and health behaviors.

### Objective

The overarching purpose of this study was to better understand DOC users and how DOC engagement is related to self-reported health outcomes. The specific aims of this exploratory and descriptive study were threefold: (1) to describe DOC users in terms of demographics, diabetes type, and diabetes-related treatment; (2) to describe intensity of use and levels of engagement of DOC users; and (3) to examine the relationship between DOC use (intensity and engagement) and health-related quality-of-life, self-care behaviors, and HbA_1c_ levels.

## Methods

### Study Design

We conducted an exploratory cross-sectional study of a convenience sample of DOC users using an online survey posted to 4 distinct DOC social media sites. First, we conducted a small pilot study of DOC users (n=5) and asked for input from 2 diabetes specialty healthcare providers to guide survey development, and to address usability and technical functionality. The final 129-item survey included questions about demographic information, health history, eHealth app use, DOC intensity and engagement, health-related quality of life (HRQoL) [46], and diabetes self-care behaviors [47]. We used Research Electronic Data Capture (REDCap) Survey software (Nashville, TN) to administer the survey. REDCap survey is a secure, Web-based study management system.

### Sample

Adult DOC users (18 years and older) with a diagnosis of type 1 or type 2 diabetes, or Latent Autoimmune Diabetes of Adulthood (LADA) who could read English, were eligible for the study. Any participant who identified themselves as having gestational diabetes, being a caregiver for someone with diabetes (ie, parent of a child with diabetes), or younger than 18 years were excluded from the analyses. The study was approved by the University of Utah Institutional Review Board, Salt Lake City, UT. The study was also approved by the administration team from TuDiabetes and Diabetic Connect; the other 2 sites were Facebook and Twitter.

### Recruitment and Setting

We recruited adult DOC users in 2 waves. We selected the initial site, TuDiabetes, because it was hosted by a nonprofit organization, the Diabetes Hands Foundation, that did not allow advertisements. TuDiabetes members were screened by an administrator before they could join, posts could be viewed by members without logging in to their account. TuDiabetes had more than 35,000 members with diabetes at the time of the study. Initially, we posted a synopsis of the study with a link to the survey on the principal investigator’s TuDiabetes profile page, which was shared by key opinion leaders and mentioned in the TuDiabetes online newsletter. The first question of the survey asked individuals if they consented to participate in the study and provided a link to further information about the study purpose and method.

A second wave of recruitment included Diabetic Connect, a for-profit organization, which was selected because of its growing diabetes-specific social media presence. Facebook and Twitter were also used for recruitment, given the number of groups, pages, and tweet chats focused on diabetes. Data collection occurred over a 7-month period.

### Measures

The online survey was divided into 6 sections: (1) demographics, (2) health history, (3) eHealth use (including reasons to join a diabetes social network, DOC intensity, DOC engagement, and internet social capital), (4) HRQoL, (5) diabetes self-care behaviors, and (6) source credibility. Moreover, 5 validated instruments were used and included the SF-12v2 [46,48], an adapted version of the Facebook intensity scale [49], an adapted version of the internet social capital scale [50], Self-Care Inventory-Revised (SCI-R) [47], and the source credibility scale [51]. This paper will examine demographics, health history, eHealth use as noted below (that includes reasons to join a DOC, DOC intensity, and DOC engagement, but does not include internet social capital), HRQoL, and diabetes self-care behaviors. Details for each measure are noted below.

#### Demographics

A total of 11 demographic items focused on gender, marital status, education level, employment, annual household income, age, ethnicity, race, country or state, living setting, and insurance status.

#### Health History

A total of 8 self-reported items focused on diabetes type, diabetes duration, current diabetes treatments, most recent HbA_1c_ level, type of medical practice, and type of healthcare provider used for diabetes care, frequency of diabetes provider visits, and presence of diabetes-related complications.

#### eHealth Use

A total of 22 items, individually scored, were asked to measure how participants navigate the DOC and if the participants’ healthcare provider knew about and supported their DOC use.

#### Reasons to Join the Diabetes Online Community

A total of 13 items were developed based on an anecdotal dLife (Diabetes Life) report [52] that addressed the reasons why someone with diabetes should join the DOC. Items were dichotomous, allowing a yes or no response.

#### Diabetes Online Community Intensity

The DOC intensity scale is an 8-item tool adapted from the Facebook intensity scale [49] to measure how often and for how long individuals are engaged in the DOC, and to determine the emotional connectedness and integration into daily activities. Scores range from 0 to 5, with higher scores indicating more DOC intensity. Cronbach coefficient for DOC intensity was .85.

#### Diabetes Online Community Engagement

The DOC engagement scale is a 5-item tool developed by the authors and informed by a qualitative analysis [36] to measure engagement or interaction with other DOC users. Specifically, this tool was used to measure whether or not participants shared clinical information, requested or provided clinical guidance or feedback, or received or provided emotional support. Scores range from 0 to 5, with higher scores indicating more DOC engagement. Cronbach coefficient for DOC engagement was .73.

#### Health-Related Quality of Life

SF12-v2 is a 12-item tool used to measure physical and mental health status. A 4-week recall was used in this study. Norm based scoring (mean 50, SD 10) was used for this analysis [46]. Cronbach coefficient for SF-12v2 was .88 (physical=.77 and mental=.86).

#### Diabetes Self-Care

SCI-R is a 15-item tool used to measure diabetes self-care behaviors and can accommodate natural variation in treatment plans for patients with type 1 and type 2 diabetes. Scores range from 0 to 100 [47]. Cronbach coefficient for the SCI-R was .68.

### Analysis

In a survey study such as ours, precision of parameter estimation is the key sample size criterion. We defined excellent precision operationally as an 80% probability of obtaining 95% confidence intervals for the mean, with half-width no greater than 0.15 SD. This criterion provides interval estimation with symmetric uncertainty that is smaller than Cohen familiar standard for a “small” effect. Under the 2-sided *t*-distribution, a sample size of 189 was required to meet this criterion, which conservatively guided our recruitment of a sample of 207. The final sample of 183 participants successfully achieved an 80.5% probability of 95% CI precision limited to 0.151 SD units.

Survey responses were identified by a participant number code, and all the study-related files were maintained in REDCap. Data were screened for multiple entries. In accordance with standard scoring methods, missing data were imputed with appropriately scaled item means in the calculation of total scores for the validated scales. All other missing data were excluded pairwise. Missing data made up less than 10% of each analysis. We performed statistical analysis using SPSS 21 (IBM) [53] and used exploratory data analysis to screen for errors, determine frequencies, and identify normality of distribution patterns. Cronbach alpha was calculated for each validated measure.

The primary goal was to gather detailed data on DOC users, both demographically and in terms of intensity and engagement in using the DOC, and to describe any relationship between DOC use and health indicators (HRQoL, self-care, and HbA_1c_ levels). To address our first aim, we ran frequencies for each demographic variable and used analysis of variance and Chi-square tests to examine if there were differences in demographic variables based on diabetes type. To address our second aim, scores for DOC intensity and DOC engagement were averaged. Analyses were conducted to determine relationships between, and interactions among, demographic variables, health history, eHealth use, DOC intensity, DOC engagement, HRQoL, and diabetes self-care behaviors, to address our third aim. This included correlations between DOC intensity, DOC engagement, HRQoL, and diabetes-self-care, as well as between the support participants received from their healthcare providers related to their DOC use, DOC intensity, and DOC engagement scores. A one sample *t* test was used to compare the studied sample with norms for diabetes related to health status [46] and diabetes self-care [47].

Variables that predicted the dichotomous outcome of HbA_1c_ <7% or ≥7%, based on the American Diabetes Association’s recommendations for an HbA_1c_ <7% [54], were examined in a simultaneous model among DOC users. To explore this, variables were analyzed based on researcher and DOC key opinion leader knowledge of the DOC in an initial stepwise logistic regression. Stepwise logistic regression allowed us to refine the variables and to remove nonsignificant variables. We then used a simultaneous logistic regression in the final predictive model. For inference, alpha was set at .05.

## Results

### Recruitment

There were 1501 unique DOC site visitors who viewed the online recruitment post and 207 unique participants who completed the survey. Of those, 183 met the inclusion criteria, giving us a recruitment rate of 12.2%. Table 1 shows participant demographic data. Participants were more likely to be female, white, living in the United States in a suburban setting, well educated, employed, and to have type 1 diabetes. Participants with type 1 diabetes were younger than those with type 2 diabetes (*P*<.001), or those with LADA (*P*=.002).

### Health History

Most of the participants reported receiving care for their diabetes at an endocrinology office (68.1%, 124/182), although those with type 2 diabetes were more likely to be seen by a family practice provider than those with type 1 diabetes (*P*<.001). Participants saw their healthcare providers at least quarterly (63.5%, 115/181) or every 6 months (23.8%, 43/181).

Participants had an average of 1.2 diabetes-related complications; there was a positive correlation between number of diabetes-related complications and diabetes duration (*r*=.369, *P*<.001). Those with type 1 diabetes were more likely to report depression (*P*=.01), heart disease (*P*=.01), and eye disease (*P*<.001) than those with type 2 diabetes or LADA. Over half (59.0%, 108/183) of individuals reported diabetes-related complications. Most commonly reported diabetes-related complications included depression (32%, 59/183), cardiovascular disease (27%, 49/183), retinopathy (21%, 38/183), and neuropathy (19%, 35/182). Diabetes treatments varied, although majority of the participants were using intensive insulin management (85%, 155/183). Of those undergoing intensive insulin management, 54.8% (81/147) were using an insulin pump, whereas 25.2% (37/147) were using a continuous glucose monitor. Respondents with type 1 diabetes had a longer diabetes duration than those with type 2 diabetes or LADA (*P*<.001).

### eHealth Use

Participants used an average of 2.6 devices to access the internet. The majority (96.2%, 175/183) of participants spent their time reading (91.3%, 167/183), responding (74.3%, 136/183), and creating original posts (59.6%, 109/183). The time for which participants had been using DOC ranged from less than 1 year (32%, 58/183) to 1 to 3 years (37.7%, 69/183), or more than 3 years (30.4%, 56/183). The majority of respondents had not told their healthcare providers about their DOC use (67.2%, 123/183). Of those who did tell their healthcare providers about their DOC use, 60% (36/60) were supported to continue doing so, 1.9% (3/183) were not supported, and 10.9% (20/183) were not sure if their provider supported their use of the DOC.

### Reasons to Join a Diabetes Online Community

A majority of the participants found participating in the DOC beneficial as it related to knowledge attainment, support, and empowerment; see Table 2. DOC users who found a benefit in their participation with the DOC reported higher DOC intensity and DOC engagement; see Table 3.

**Table 1:** *.* Demographics by type of diabetes.

Characteristics		Type 1 diabetes (n=129)	Type 2 diabetes (n=33)	LADA^a^ (n=21)	Total	*P* value
Age in years, mean (SD)^b^		41 (13.6)	51.2 (11.4)	52.6 (13.7)	44.7 (14.0)	<.001
Diagnosis (duration in years), mean (SD)^b^		22.5 (14.6)	6.4 (5.7)	10.4 (10.2)	18.2 (14.6)	<.001
**Gender, n (%)^c^**						.09
	Male	31 (24.4)	14 (42.4)	4 (20)	49 (26.8)	
	Female	96 (75.6)	19 (57.6)	16 (80)	131 (71.6)	
**Ethnicity, n (%)^c^**						.27
	Hispanic or Latino	6 (4.7)	0 (0.0)	0 (0)	6 (3.3)	
	Not Hispanic or Latino	121 (95.3)	33 (100)	21 (100)	175 (95.6)	
**Race, n (%)^c^**						.73
	American Indian or Alaskan Native	2 (1.6)	0 (0.0)	0 (0)	2 (1.1)	
	Asian	2 (1.6)	0 (0.0)	1 (5)	3 (1.6)	
	African American	2 (1.6)	0 (0.0)	0 (0)	2 (1.1)	
	White	122 (95.3)	33 (100.0)	19 (95)	174 (95.1)	
**Country, n (%)^c^**						.64
	United States	108 (84.4)	27 (81.8)	16 (76.2)	151 (82.5)	
	Not United States	20 (15.6)	6 (18.2)	5 (24)	31 (16.9)	
**Living setting, n (%)^c^**						.03
	Rural	16 (12.4)	11 (33.3)	6 (28.6)	33 (18.0)	
	Suburban	78 (60.5)	16 (48.5)	8 (38.1)	102 (55.7)	
	Urban	35 (27.1)	6 (18.2)	7 (33.3)	48 (26.2)	
**Income, n (%)^c^**						.58
	Less than US $30,000	28 (23)	12 (36.4)	3 (16.7)	43 (23.5)	
	US $30,000-$49,999	20 (16.4)	5 (15.2)	5 (27.8)	30 (16.4)	
	US $50,000-$74,999	24 (19.7)	6 (18.2)	4 (22.2)	34 (18.6)	
**Education, n (%)^c^**						.001
	Some high school	2 (1.6)	0 (0.0)	0 (0)	2 (1.1)	
	High school graduate	5 (3.9)	6 (18.2)	0 (0)	11 (6.0)	
	Some college	13 (10.2)	7 (21.2)	8 (38.1)	28 (15.3)	
	Associate’s degree	11 (8.6)	6 (18.2)	3 (14.3)	20 (10.9)	
	Bachelor’s degree	54 (42.2)	8 (24.2)	4 (19)	66 (36.1)	
	Graduate degree	43 (33.6)	6 (18.2)	6 (28.6)	55 (30.1)	
**Employment, n (%)^c^**						.19
	Student	12 (9.3)	2 (6.1)	1 (5)	15 (8.2)	
	Unemployed	8 (6.2)	5 (15.2)	1 (5)	14 (7.7)	
	Working part-time	20 (15.5)	4 (12.1)	3 (15)	27 (14.8)	
	Working full-time	67 (51.9)	13 (39.4)	10 (50)	90 (49.2)	
	Retired	9 (7)	6 (18.2)	5 (25)	20 (10.9)	
	Disabled	13 (10.1)	3 (9.1)	0 (0)	16 (8.7)	
**Insurance, n (%)^c^**						.63
	Insured	111(92.5)	32(97)	19 (95)	162 (88.5)	
	Uninsured	9 (7.5)	1 (3)	1 (5)	11 (6.0)	
**Treatment, n (%)^c^**						<.001
	No medications	0 (0)	3 (10)	0 (0)	3 (1.6)	
	Oral agents only	0 (0)	15 (45)	0 (0)	15 (8.2)	
	One injection^d^	0 (0)	7 (21)	3 (14)	10 (5.5)	
	Intensive insulin	129 (100)	8 (24)	18 (86)	155 (84.7)	
**Type of practice, n (%)^c^**						<.001
	Endocrinology	101 (78)	8 (24)	15 (75)	134 (67)	
	Internal medicine	14 (11)	8 (24)	0 (0)	22 (11.9)	
	Family practice	12 (7)	15 (46)	3 (15)	30 (16.2)	
	Community clinic	2 (2)	1 (3)	1 (5)	4 (2.2)	
	Other	0 (0)	1 (3)	1 (5)	2 (1.1)	

^a^Latent autoimmune diabetes of adulthood.

^b^Analysis of variance.

^c^Chi-square.

^d^One injected medication (ie, basal insulin, incretin mimetic) with or without oral medications.

**Table 2:** *.* Reasons to join a diabetes online community (DOC); N ranges from 169 to 176.

Reason to join a DOC	n (% stating yes)
The DOC helped me learn research and treatment alternatives	146 (83.4)
The DOC allows me to help others	142 (80.9)
The DOC helped me learn new diabetes management strategies	139 (80.3)
The DOC helps me feel understood	138 (79.3)
The DOC helped me get answers to many of my diabetes questions	133 (76.0)
The DOC helps me feel less alone	128 (75.7)
The DOC helps me feel more empowered	128 (73.1)
The DOC allows me to make new friends	113 (66.1)
The DOC helped me learn about potential side effects of drugs or devices	112 (64.0)
The DOC helped me learn things that my healthcare provider did not know	102 (60.0)
The DOC helps me feel support through rough times	99 (57.9)
The DOC helped me learn strategies to improve insurance coverage for diabetes-related medications or supplies	84 (47.7)
I discussed a topic I learned about on the DOC with my healthcare provider	82 (48.5)

**Table 3:** Relationship between diabetes online community (DOC) benefits, intensity, and engagement; N ranges from 169 to 176.

DOC benefit		DOC intensity		DOC engagement	
		Mean (SD)	*P* value	Mean (SD)	*P* value
**Feel understood**			<.001		<.001
	Yes	3.0 (0.65)		2.7 (1.6)	
	No	2.1 (0.64)		1.2 (1.3)	
**Feel less alone**			<.001		<.001
	Yes	3.0 (0.62)		2.7 (1.7)	
	No	2.2 (0.60)		1.4 (1.2)	
**Feel more empowered**			<.001		<.001
	Yes	3.0 (0.63)		2.8 (1.6)	
	No	2.1 (0.60)		1.0 (1.1)	
**Feel support through rough times**			<.001		<.001
	Yes	3.1 (0.62)		2.92 (1.6)	
	No	2.4 (0.70)		1.57 (1.5)	
**Learn new diabetes management strategies**			<.001		<.001
	Yes	2.9 (0.67)		2.6 (1.6)	
	No	2.1 (0.64)		1.4 (1.4)	
**Learn research and treatment alternatives**			<.001		<.001
	Yes	2.9 (0.66)		2.6 (1.7)	
	No	2.1 (0.70)		1.0 (0.98)	
**Get answers to diabetes questions**			<.001		<.001
	Yes	3.0 (0.65)		2.7 (1.6)	
	No	2.2 (0.67)		1.4 (1.4)	
**Learn about potential side effects of drugs or devices**			<.001		<.001
	Yes	3.0 (0.65)		2.8 (1.6)	
	No	2.3 (0.68)		1.5 (1.4)	
**Learn things that my healthcare provider didn’t know**			<.001		<.001
	Yes	3.0 (0.68)		2.8 (1.6)	
	No	2.4 (0.70)		1.5 (1.4)	
**Learn strategies to improve insurance coverage for diabetes-related medications or supplies or tools**			<.001		.003
	Yes	3.0 (0.66)		2.7 (1.7)	
	No	2.5 (0.72)		2.0 (1.6)	
**Discussed a topic learned from DOC with my healthcare provider**			<.001		<.001
	Yes	3.0 (0.68)		3.2 (1.6)	
	No	2.5 (0.68)		1.6 (1.4)	
**Help others**			<.001		<.001
	Yes	2.9 (0.66)		2.7 (1.6)	
	No	2.1 (0.74)		0.70 (0.88)	

### Diabetes Online Community Intensity

The average DOC intensity scale score was 2.76 (SD .73) on a scale of 0 to 5. There was a difference in the intensity with which participants were using the DOC when comparing the 4 diabetes treatments (*F*_3,177_=3.5, *P*=.02). Respondents who were on no medications (mean 3.1, SD 0.80) or on intensive insulin management (mean 2.8, SD 0.71) had higher DOC intensity scores when compared with those taking oral agents only (mean 2.3, SD 0.69). DOC intensity scores varied based on whether or not DOC users had told their healthcare providers about their DOC use, and if it was supported (*F*_3,170_=11.3, *P*<.001). Specifically, DOC intensity scores were higher in those participants who had told their healthcare providers about their DOC use and felt supported (mean 3.2, SD 0.64) or were not sure (mean 3.2, SD 0.57) than those who had never told their healthcare providers about their DOC use at all (mean 2.6, SD 0.71). Type of diabetes or length of time using the DOC was not associated with DOC intensity. DOC intensity and DOC engagement were positively correlated (*r*=.572, *P*<.001).

### Diabetes Online Community Engagement

The average DOC engagement score was 2.24 (SD 1.69) on a scale of 0 to 5. DOC engagement scores were related to healthcare provider knowledge and support of DOC use (*F*_3,170_=11.0, *P*<.001). DOC engagement scores were higher for those who had told their healthcare providers about their DOC use and were unsure if they were supported (mean 2.9, SD 1.3) or felt supported (mean 3.6, SD 1.4) than for those who had never told their healthcare providers about their DOC use at all (mean 1.9, SD 1.6). DOC engagement scores were higher the longer someone had participated in the DOC. Those who had participated in the DOC for 4 or more years (mean 2.86, SD 1.7) were more engaged than those who had participated for less than 3 months (mean 1.50, SD 1.5, *P*<.001). There was no difference in DOC engagement scores for those who were insured or uninsured or by type of diabetes. Furthermore, there was no correlation between DOC engagement and age, diabetes type, or diabetes duration.

### Health-Related Quality of Life

The SF-12v2 physical component summary mean score was 64.8 (SD 19) and the mental component mean summary score was 66.57 (SD 21.1); both were higher (*P*<.001, one sample *t* test) than previously reported physical component summary norms of individuals with diabetes [46]. The SF-12v2 physical component summary score negatively correlated with age (*r*=−.177, *P*=.02). The physical component summary and mental component summary were not related to diabetes type, DOC engagement, and DOC intensity.

### Diabetes Self-Care Behaviors

On average, DOC participants had high self-care scores (mean 72.4, SD 12.0) compared with mean scores found in other samples of adults with type 1 and type 2 diabetes (*P*<.001, one-sample *t* test) [47]. Diabetes self-care behavior scores were lower in those who reported depression (reported depression mean 68.9, SD 13.8; reported no depression mean 74.1, SD 10.8, *P*=.007). There were positive correlations between self-care scores and DOC engagement scores (*r*=.170, *P*=.02), DOC intensity scores (*r*=.236, *P*=.002), and SF-12v2 mental component summary scores (*r*=.301, *P*<.001). There was a negative correlation between self-care scores and HbA_1c_ (*r*=−.157, *P*=.04). Correlation between diabetes self-care, HRQoL, DOC intensity, and DOC engagement is noted in Table 4.

### Glycated Hemoglobin A1c Levels and Predictors

The majority (59.6%, 109/183) of survey respondents reported an HbA_1c_<7%. There was no difference in HbA_1c_ levels between US users and non-US users, insured and uninsured users, or type of diabetes. After conducting an initial stepwise logistic regression, the final predictive binary logistic regression model (see Table 5) was employed to explain the HbA_1c_ category of <7% or ≥7% while controlling for all other variables in the model. The odds ratio for age was significant, with every 1-year increase in age yielding 34% reduction in the odds of having an HbA_1c_≥7%. Diabetes duration generated a 1.46 odds ratio of having an HbA_1c_≥7%. DOC engagement was a strong predictor of HbA_1c_ level; every single point increase in DOC engagement yielded a 33.8% reduction in the odds of an individual having an HbA_1c_≥7%. There was a 2.7 times increase in the odds of having an HbA_1c_≥7% among participants who reported that DOC helped them learn about strategies to improve insurance coverage for diabetes-related medications, supplies, and technology devices (coded yes or no).

**Table 4:** Correlation matrix for health indicators.

Health Indicator		1	2	3	4	5
1	DOC^a^ intensity	1.00				
2	DOC engagement	.572^c^	1.00			
3	Physical HRQoL^b^	−.043	.102	1.00		
4	Mental HRQoL	−.076	.074	.651^c^	1.00	
5	Diabetes self-care	.236^d^	.170^e^	.097	.301^d^	1.00

^a^DOC: diabetes online community.

^b^HRQoL: health-related quality of life.

^c^Significance at the <.001 level.

^d^Significance at the <.01 level.

**Table 5:** Final model explaining risk of glycated hemoglobin A_1c_≥7%.

Variable	Beta	SE	*P* value^a^	Exp (B)=odds ratio	95% CI for Exp (B)
DOC^b^ engagement^c^	−.413	.132	.002	0.662	0.511-0.857
Diabetes diagnosis duration^d^	.377	.108	<.001	1.459	1.180-1.803
Learned insurance coverage strategies	.987	.406	.02	2.684	1.212-5.944
Help others	−.952	.557	.09	0.386	.0130-1.150
Support through rough times	.808	.441	.07	2.243	0.946-5.320
Age in years	−.035	.014	.01	.966	0.940-0.992
Constant	.349	.793	.66	1.417	

^a^*P* value of Wald ratio.

^b^DOC: diabetes online community.

^c^Mean score of 5 dichotomous variables, coded 0 to 5.

^d^Length of time in years since diabetes diagnosis using a square root transformation to address a positive skew.

## Discussion

The purpose of this study was to explore who uses the DOC, how they use it, and whether DOC use is associated with specific health indicators. Below we discuss the significant findings that support both the importance of the DOC for specific populations with diabetes and the positive association of DOC use with health indicators. We also discuss implications for clinical practice.

### Principal Findings

We found an overwhelming representation of type 1 diabetes within this sample of DOC users, even though type 1 diabetes makes up only 5% to 10% of all diagnosed cases of diabetes [55]. This overrepresentation may be due to the fact that one of our recruitment sites, TuDiabaetes, had mostly individuals with type 1 diabetes using their website. An alternative explanation is that individuals with type 1 diabetes must utilize intensive insulin management techniques, whereas individuals with type 2 diabetes may not. Intensive insulin management may drive an additional need for knowledge and support, leading patients to DOC. Furthermore, those with type 1 diabetes have more exposure to technology, given that they typically are diagnosed much younger and typically use a glucometer. Those with type 1 diabetes in this sample were younger and potentially more likely to use social media in general [56]. Finally, because there are fewer individuals with type 1 diabetes compared with type 2 diabetes in the general population, those with type 1 diabetes may not be able to connect with another person with their same condition offline and this may lead them to seek others like themselves online [2]. Diabetes research conducted in other online communities, such as PatientsLikeMe, have found more respondents with type 2 diabetes, suggesting that other factors such as DOC site user characteristics and site purpose may influence who participates [57]. There was no significant difference between type of diabetes as it related to DOC engagement or HbA_1c_.

The majority of the participants had not told their healthcare providers about their DOC use. Although our findings support the idea that DOC use is supplementary to, not in place of, regular healthcare provider visits, research has shown that healthcare providers may be hesitant to suggest DOC use due to concerns about misinformation [58,59], fear of a power imbalance from the traditional hierarchy of medicine [60,61], or fear of a challenge to their authority [62]. It is important for healthcare providers to be aware of the DOC and how health-related social media is driving a more patient-centered healthcare system [63,64] by putting the patient’s preferences and values about how they want to receive healthcare front and center [65], consistent with the eHealth-enhanced chronic care model [66]. Furthermore, healthcare providers should be learning how they can engage with DOC themselves while supporting their patients with diabetes to use the DOC [28,31,67]. If healthcare providers discuss and support DOC use with their patients, patients may be encouraged to access quality online diabetes self-care information and support. In this way, the DOC could be a complementary resource for information to support health literacy not found in the traditional healthcare model.

DOC intensity varied by treatment. Those with no medications or on intensive insulin management used DOC more intensely than those on oral agents. This is perhaps due to the fact that individuals on no medications may be accessing the DOC to educate themselves with hopes of halting the progression of their diabetes, whereas those on intensive insulin management require more education, skills, and support to manage their diabetes than those on oral agents only. Similar to other research, our study found associations between intensity of DOC use and feeling supported in disease management [68]. We did not find differences in DOC intensity between insured and uninsured participants; conversely, other research has shown that individuals with chronic conditions who were uninsured were more likely than those who were privately insured to be frequent users of online health information [69].

Our research indicates that DOC users have higher HRQoL when compared with HRQoL norms for the general population [46]. Individuals who seek online health information report being happier and healthier when compared with those who seek offline health information [70]. DOC users can quickly access health-related information they desire in multiple formats (ie, discussion board, blog, Tweetchat, etc), allowing them to easily review crowdsourced information from individuals living with diabetes, learn the same information in a variety of ways [8] from different DOC users to address learning style preferences, and focus on topics based on need and interest. The ability to obtain health information from the DOC in multiple ways supports a patient-centered approach to enhancing health literacy. The DOC also provides an avenue for individuals with diabetes to provide social support to one another. Social support, which has been linked to HRQoL scores [71], allows individuals to feel less alone in their diabetes. Individuals reported a sense of social connectedness, which strongly predicts altruism [72]. Altruism has been identified as a factor in participating in chronic disease online communities [73-75], which may enhance the social learning process.

This is the first study to demonstrate that engaging in the DOC is associated with positive health benefits for people with diabetes. DOC engagement is related to better glycemic levels, diabetes self-care, and HRQoL. DOC engagement allows individuals to share personal experiences, exchange emotional support, and gain expertise in day-to-day management techniques through crowdsourced information by peers. Although it is important to note that directionality and causation cannot be determined in this model, there is evidence to suggest that DOC engagement may lead to improved HbA_1c_ levels. Individuals who have an HbA_1c_≥7% and longer diabetes duration may be engaging in the DOC to connect with others due to diabetes burnout. Furthermore, individuals who already had an HbA_1c_≥7% may have sought support from the DOC to learn strategies to improve insurance coverage of diabetes-related expenses so they could in turn improve their diabetes management. Longitudinal research is necessary to understand glycemic levels as it relates to specifics of DOC use, such as learning how to improve insurance coverage for diabetes-related expenses.

Individuals with diabetes who are actively engaging in the DOC are actively participating in their own healthcare. Patient activation, known to decrease healthcare costs, is gauged by knowledge, skills, and confidence one has to manage his or her own health [76], which is associated with engagement in online communities [77]. In this study, DOC engagement was associated with increasing diabetes-related knowledge and skills, self-care, and empowerment, supporting the notion of high patient activation. Health literacy may also improve with increased diabetes-related knowledge. Research has shown that the interaction between patient activation and health literacy is associated with better glycemic levels [78]. Furthermore, patients who actively participate in medical decisions have improved glycemic levels [79]. Additional research is needed to distinctly identify how DOC impacts glycemic levels, patient activation, and health literacy.

DOC engagement was higher for DOC users whose healthcare providers supported them in their DOC use. Although it is important to note that a causal inference cannot be made, this finding has potential clinical implications in that DOC engagement may supplement current diabetes care and lead to improved glycemic levels. Participation in the DOC requires no resource allocation from the current healthcare system, although it is only available to those with internet access and a sufficient level of health literacy to use it. Despite documented benefits of face-to-face peer health [11-13,80], there are currently no professional recommendations for individuals to use peer health via the DOC to supplement their diabetes care.

### Limitations

We recruited our sample from the DOC, and therefore, caution must be exercised when generalizing because of the possibility of bias due to sample self-selection. Individuals who responded to the survey may be more engaged with the DOC or have better glycemic levels. The majority of individuals in this sample identified themselves as using intensive insulin management, which does not reflect the treatment intensity seen in the general population. We had a response rate of 12.2% based on the number of times the study recruitment post was viewed by unique site visitors. Although the recruitment percentage may appear low, a response rate of <.1% is not unusual for online surveys [81]. The respondents were overwhelmingly white, college-educated females living in the United States, which may not be an accurate reflection of the entire DOC population, and is not an accurate reflection of the general population with diabetes. For example, individuals who are American Indian or Alaskan Native, black, and Hispanic are more likely to have diabetes than those who are white [82]. Finally, this study only looked at adult DOC users, and findings should not be generalized to individuals with gestational diabetes, minors, or caregivers participating in the DOC.

Self-reporting of HbA_1c_ may affect reliability of data; however, research that validated self-reported HbA_1c_ with laboratory values has shown that self-reported diabetes data are accurate >92% of the time [83]. Similar HbA_1c_ results have been found among international DOC users, in which the average HbA_1c_ was 6.9% [84]. In addition, some DOC participants have been found to share their HbA_1c_ levels with others online [84], and have gone as far as including a photograph of their lab record. Transparency in sharing health information, as seen in recent #wearenotwaiting and #OpenAPS movements on Twitter and other social media sites [85], may improve reliability in reporting, although we did not request HbA_1c_ documentation for this study.

The nature of this research cannot determine causality. We do not know if the high DOC engagement results in high self-care and optimal glycemic levels, or vice versa, or if common unknown causal factors induce the association. Prospective studies, specifically randomized control trials, are warranted to better understand DOC and its impact on health outcomes.

### Conclusions

Our study found that higher engagement with the DOC was associated with HbA_1c_ levels <7%, although we cannot determine directionality of this relationship. We also found that DOC users are generally proactive in diabetes self-care behaviors and that there was a strong sense of community among DOC participants. Participants found DOC peer health to be beneficial with regard to knowledge attainment and support, factors known to enhance health literacy. Our survey indicated that DOC users are often not informing their healthcare providers about their participation with the DOC. Our findings suggest that healthcare providers should be familiar with DOC and ask their patients about use of online sources for diabetes self-care information and support. Supplementing usual healthcare activities with DOC use may encourage knowledge and support among a population that can benefit greatly from optimizing diabetes self-care. This study adds to the body of knowledge in diabetes care and online communities for chronic disease management. Further studies to determine how DOC use affects health outcomes, and how health behaviors contagiously spread throughout the DOC, would be enlightening.

## Abbreviations

DOC: diabetes online community
HRQoL: health-related quality of life
LADA: Latent Autoimmune Diabetes of Adulthood
REDCap: Research Electronic Data Capture
SCI-R: Self-Care Inventory-Revised
